# Interleukin-5, Eosinophil, and Immunoglobulin A Levels in Schizophrenia Patients

**DOI:** 10.31083/AP46059

**Published:** 2025-08-26

**Authors:** Xi Li, Xiaoyu Wang, Qianqian Zhou, Qiushan Zhang, Shujuan Pan

**Affiliations:** ^1^Department of Clinical Laboratory, Beijing Huilongguan Hospital, 100096 Beijing, China

**Keywords:** schizophrenia, interleukin-5, eosinophils, immunoglobulin A

## Abstract

**Objective:**

To analyze the correlation between interleukin-5 (IL-5), eosinophils (EOS), and immunoglobulin A (IgA) levels with schizophrenia, and assess their potential as auxiliary diagnostic markers for schizophrenia.

**Methods:**

This study comprised 57 patients with first-episode schizophrenia and 340 patients with recurrent or chronic schizophrenia who were hospitalized at Beijing Huilongguan Hospital from March 2023 to August 2024, and 72 healthy volunteers were recruited as the control group. Fasting venous blood samples were collected from all participants on the second day after admission. For patients with first-episode schizophrenia, a second blood draw was performed after two months of treatment. Simultaneously, the Positive and Negative Symptom Scale (PANSS) was administered to assess the subjects. IL-5 and EOS levels were measured using flow cytometry; IgA levels were measured using immunoturbidimetry. SPSS v.29.0 was used to conduct *t*-tests, one-way ANOVA, correlation analysis and receiver operating characteristic (ROC) curve analysis.

**Results:**

The first-episode schizophrenia group and the recurrent/chronic schizophrenia group had elevated IL-5 levels relative to healthy controls; however, the increase in EOS levels was specifically observed in the recurrent/chronic schizophrenia group. After treatment, the IL-5 level in the first-episode group was markedly reduced. Correlation analysis revealed that in patients with schizophrenia, IL-5 levels were positively correlated with EOS (r = 0.338, *p* < 0.001), and EOS levels were positively associated with disease duration (r = 0.171, *p* < 0.05), the ROC curve analysis revealed that IL-5 had a sensitivity of 52.9%, specificity of 69.4%, and a cut-off value of 2.445 pg/mL for predicting schizophrenia.

**Conclusion:**

In patients with schizophrenia, the elevated levels of IL-5 and EOS appear to be disease-related rather than medication-induced, suggesting their potential involvement in the inflammatory pathogenesis of schizophrenia. Furthermore, IL-5 exhibits greater predictive accuracy for schizophrenia compared to EOS, suggesting that IL-5 may serve as a valuable biomarker for auxiliary diagnosis and stratification analysis in schizophrenia.

## Main Points

∙ IL-5 levels were significantly elevated in both the first-episode schizophrenia group and the recurrent/chronic schizophrenia group, whereas eosinophils (EOS) levels were exclusively elevated in the recurrent/chronic schizophrenia group. The observed increase in eosinophils may be associated with increased IL-5 levels, and patients with a longer disease duration tended to exhibit higher eosinophil counts.

∙ Following two months of treatment, Positive and Negative Symptom Scale (PANSS) scores and IL-5 levels in patients with first-episode schizophrenia decreased significantly, suggesting that the elevation in IL-5 is more likely attributable to the disease itself rather than medication effects.

∙ IL-5 demonstrates greater predictive accuracy for schizophrenia compared to EOS; however, its diagnostic value as a standalone marker remains limited.

## 1. Introduction

Schizophrenia is a serious mental condition characterized by positive symptoms (e.g., delusions and hallucinations), negative symptoms (e.g., anhedonia and social withdrawal), affective symptoms, and cognitive dysfunction [1, 2]. The course of the illness is generally prolonged, with a tendency for relapse that can gradually progress to chronic deterioration of mental function. This condition inflicts varying degrees of harm on affected individuals, their families, and society [3, 4]. The exact pathogenesis of schizophrenia remains incompletely understood, although it is thought to be related to the interplay between environmental and genetic factors [5, 6]. Numerous hypotheses have been proposed regarding its etiology, including neurodevelopmental theories and neurochemical hypotheses associated with neurotransmitters, such as dopamine, serotonin, and glutamate [7, 8]. In recent years, accumulated evidence from an increasing amount of research has suggested that the onset and progression of schizophrenia are linked to immune inflammation [9].

Inflammation can lead to negative emotions and anxiety, as well as impact cognitive processes. Existing research indicates that pro-inflammatory cytokines in the central nervous system may contribute to increased depressive behaviors; neuro-inflammation enhanced by the activation of microglia (immune effector cells within the brain) affects endothelial cells of the blood-brain barrier and promotes the recruitment and transport of peripheral immune cells to stress-sensitive neural regions, which can also cause behavioral changes; sustained inflammation during brain development can potentially interfere with normal brain maturation [7, 10, 11]. In the study of schizophrenia, some patients exhibit varying degrees of systemic inflammatory states, specifically evidenced by enhanced activation of microglia in the brain, increased levels of pro-inflammatory cytokines such as interleukin-1β (IL-1β), interleukin-6 (IL-6), and tumor necrosis factor α (TNF-α), as well as increased expression of membrane-bound receptors for pro-inflammatory cytokines, such as interleukin-1 receptor type 1 (IL-1R1), tumor necrosis factor receptor 1 (TNFR1), and tumor necrosis factor receptor 2 (TNFR2). Activated microglia release cytokines that exert pro-inflammatory effects through binding to specific receptors, prolonged inflammation may contribute to neuronal damage in the brain [11, 12, 13, 14, 15, 16].

The administration of anti-inflammatory agents (e.g., non-steroidal anti-inflammatory drugs and N-acetylcysteine) has been reported to mitigate the severity of schizophrenia symptoms. Anti-psychotic medications have been shown to reduce the over-activated inflammatory response observed in psychiatric conditions. For example, haloperidol, risperidone, and aripiprazole effectively suppress the expression of pro-inflammatory markers on microglial cells, including CD16/32 and CD86; aripiprazole increases the expression of anti-inflammatory markers, such as CD206 and Arg-1, leading to reduced levels of pro-inflammatory cytokines (e.g., IL-1β, TNF-α and IL-6) [17, 18]. Persistent neuroinflammation is therefore considered to be a potential mechanism underlying the onset and progression of schizophrenia, and the alleviation of inflammatory responses is a promising therapeutic approach that merits further investigation.

IL-5 is a homodimeric glycoprotein secreted by T-helper type 2 (Th2) cells upon stimulation. This cytokine exerts its effects on target cells by binding to its specific receptor, IL-5R, which consists of a unique α chain (IL-5Ra) and a common β chain (βc), IL-5Ra specifically binds to IL-5, while βc serves as a signaling molecule common to IL-3R and granulocyte-macrophage colony-stimulating factor receptor (GM-CSFR). Both B cells and eosinophils (EOS) express IL-5Ra and respond to IL-5 by activating pathways such as the janus kinase/signal transduction and activator of transcription (JAK/STAT) and Ras-extracellular signal-regulated kinase pathways, thereby promoting the proliferation, differentiation, and survival of these cells. Immunoglobulin A (IgA) represents the predominant antibody type in the human body, IL-5 and IL-6 can drive the differentiation of B cells into plasma cells, leading to increased secretion of antibodies, and in particular IgA and IgM [19, 20, 21].

Research on IL-5 has revealed that the colony-stimulating factor 2 receptor beta (*CSF2Rβ*) gene is located in a linkage region associated with schizophrenia, *CSF2Rβ* encodes the protein which is the common β chain of the high affinity receptor for interleukin-3 (IL-3), IL-5 and colony-stimulating factor (CSF). Single nucleotide polymorphisms (SNPs) in *CSF2Rβ* are considered to be associated with schizophrenia and depression [22]. Several studies have reported elevated IL-5 levels in patients with depression, and demonstrated that both IL-5 and GM-CSF levels significantly decrease following antidepressant treatment [23, 24]. Research on EOS has revealed that eosinophil chemotactic factor 1 (CCL11) is closely associated with aging, impaired neurogenesis, and neurodegenerative diseases. Notably, CCL11 levels are elevated in patients with schizophrenia, major depressive disorder, and bipolar disorder, with this alteration exhibiting similarities across different psychiatric conditions [25]. However, it has been observed that patients with untreated major depressive disorder exhibit reduced EOS levels. Following treatment, EOS levels tend to increase, potentially attributable to the immunosuppressive effects of the stress hormone cortisol [26]. Collectively, these findings indicate that IL-5 and EOS may be involved in the pathogenesis and progression of mental disorders. Current research on cytokines related to schizophrenia has primarily focused on TNF-α, IL-1β, and IL-6 [27, 28]. Research on IL-5 with schizophrenia has so far been limited, and the mechanisms underlying its potential role remain unclear. Therefore, the aim of this study was to provide new evidence to enable a better understanding of the roles of IL-5, EOS, and IgA in the pathogenesis and progression of schizophrenia.

## 2. Methods

### 2.1 Data Collection

Patient data was collected from March 2023 to August 2024 at the Huilongguan Hospital in Beijing. The study included 57 first-episode schizophrenia patients who had not received systematic pharmacological treatment, 340 patients with recurrent or chronic schizophrenia, and 72 healthy controls. The inclusion criteria were: (1) diagnosis of schizophrenia made by two senior physicians based on the relevant standards of the International Classification of Diseases, Tenth Revision (ICD-10); (2) patients had not recently used immunosuppressants, antibiotics, chemotherapeutic agents, or other medications that affect immune status; (3) no infections occurred within one month prior to inclusion. The exclusion criteria were: (1) presence of other severe neurological disorders; (2) existence of serious somatic disease; (3) female participants who were pregnant or breastfeeding; (4) patients who developed infections during the data collection period while hospitalized. The study was approved by the Ethics Committee of Beijing Huilongguan Hospital.

### 2.2 Measurements

#### 2.2.1 Patient Grouping and Sample Collection

Blood was collected from all study subjects on the second morning of hospitalization after fasting. The 397 schizophrenia patients were categorized into two groups: 57 first-episode cases, and 340 recurrent or chronic schizophrenia cases. Additionally, blood for follow-up testing was collected from first-episode patients on the morning after a two-month systematic treatment regimen.

#### 2.2.2 Measurement of IL-5

Three mL of venous blood was collected from each patient in a fasting state and transferred to a tube containing 10% ethylenediaminetetraacetic acid (EDTA) anticoagulant (111030049, IMPROVE Medical Corp, Zhuhai, Guangdong, China). The sample was gently mixed by inversion and then centrifuged at 1000 ×g for 20 minutes to separate the plasma, which was subsequently stored at –80 °C for future analysis. The measurement of cytokine was carried out using a multiplex, microsphere-based flow cytometric immunoassay. The Agilent NovoCyte D2060R flow cytometer (Agilent Tech Corp, Hangzhou, Zhejiang, China) was employed as the analytical instrument, and the RAISECARE cytokine detection kit (R6401002, RAISECARE Biotech Corp, Qingdao, Shandong, China) was utilized as the reagent. The detailed procedure is described below. Initially, 25 µL of plasma sample and 25 µL of buffer solution were added to the sample tube. Next, 25 µL of capture microsphere antibody and 25 µL of detection antibody were introduced, followed by incubation in the dark at room temperature and with shaking at 400–500 r/min for 2 h. Thereafter, 25 µL of streptavidin-phycoerythrin (SA-PE) was added, and the mixture was further incubated under the same conditions for an additional 0.5 h. Finally, 1000 µL of washing buffer was added, and the sample was vortexed and centrifuged at 300–500 g for 5 minutes. The supernatant was carefully discarded, and the sample tube was inverted on absorbent paper to remove residual liquid. Lastly, 200 µL of washing buffer was added to resuspend the pellet, and the sample was analyzed using flow cytometry.

#### 2.2.3 Measurement of IgA

Five mL of venous blood was collected from each patient in a fasting state and placed in a plain tube without anticoagulant (111150147, IMPROVE Medical Corp). The sample was centrifuged at 3000 ×g for 15 minutes at 4 °C to separate the serum, which was then stored at –80 °C for subsequent analysis. The detection of IgA was performed using the nephelometric turbidimetry method, the IMMAGE 800 Special Protein Analyzer (A15445, Beckman Coulter Corp, Brea, CA, USA) was employed as the analytical instrument, and the Beckman IgA Test Kit (446460, Beckman Coulter Corp) was utilized as the reagent. The serum supernatant was collected for on-instrument analysis. The detection principle relies on the enhanced scattered light intensity resulting from the formation of antigen-antibody complexes. Quantitative analysis of IgA was achieved by measuring the change in scattered light intensity within the reaction well.

#### 2.2.4 Measurement of EOS

Three mL of venous blood was collected from each patient in a fasting state and transferred to a tube containing 10% EDTA anticoagulant. Immediate testing of the sample was performed following collection. The quantification of EOS was performed using a semiconductor laser flow cytometry method. A Sysmex XN-3000 hematology analyzer (SYSMEX Corp, Kobe, Japan) was employed as the analytical instrument, with reagents provided by Sysmex. The sample was gently mixed and then tested on the instrument. EOS were quantitatively analyzed by detecting the intensity of side-scattered light and side fluorescence.

#### 2.2.5 Method for Assessing the Severity of Clinical Symptoms 

On the day of blood collection, the severity of patients’ clinical symptoms was quantitatively assessed using the Positive and Negative Syndrome Scale (PANSS). The PANSS comprises three subscales totaling 30 items: the Positive Symptom Subscale (7 items), the Negative Symptom Subscale (7 items), and the General Psychopathology Subscale (16 items). Each item is rated on a 7-point scale ranging from 1 to 7, with an overall score range of 30 to 210. Higher scores correspond to more severe clinical symptoms.

### 2.3 Statistical Analysis

Statistical analyses were performed using SPSS software (version 29, IBM Corp, Armonk, NY, USA). For comparisons between groups, continuous variables were first assessed for normality. If normally distributed, they were presented as $\bar{x}$ ± sd, with the *t* test used for two group comparisons. Analysis of variance (ANOVA) was employed to compare differences between multiple groups, with pairwise comparisons performed using the Bonferroni test or Games-Howell test. If the data did not meet normality assumptions, they were presented as M (QR), with the Kruskal-Wallis test used for multiple group comparisons. Categorical variables compared between groups using the χ^2^ test. Partial correlation analysis was performed to evaluate the relationships among variables, with Bonferroni correction applied to control for multiple comparisons. The statistical significance level was set at *p* < 0.05.

## 3. Results

### 3.1 Elevated IL-5 and EOS Levels in Patients With Schizophrenia

A total of 397 patients with schizophrenia were enrolled in this study, comprising 200 males (50.38%) and 197 females (49.62%). No significant differences in the gender ratio or age distribution were observed between the schizophrenia and the healthy control groups. The results showed that IL-5 and EOS levels were significantly higher in the schizophrenia group (*p* < 0.05), but no significant difference was found in the IgA level (*p* > 0.05). Subgroup analyses based on age, gender, and disease severity further revealed that IL-5 levels were significantly elevated in schizophrenia patients compared to healthy controls across all subgroups, except for those aged >60 years (Table 1). Moreover, no significant variations in IL-5 levels were detected across groups categorized by age, gender, or symptom severity.

**Table 1.** **IL-5, EOS, and IgA levels in patients with schizophrenia and in healthy individuals**.

		Patients with schizophrenia		Healthy individuals		*p*
		N	$\bar{x}$ ± sd	N	$\bar{x}$ ± sd	
IL-5 (pg/mL)		397	2.88 ± 1.51	72	2.26 ± 1.06	<0.001^**^
Age		*p* = 0.373		*p* = 0.435		
	≤30 years	77	2.88 ± 1.20	19	2.24 ± 1.17	0.039^*^
	≤45 years	116	2.79 ± 1.54	20	2.28 ± 0.86	0.041^*^
	≤60 years	138	3.05 ± 1.63	19	2.27 ± 1.13	0.048^*^
	>60 years	66	2.69 ± 1.50	14	2.23 ± 1.17	0.284
Gender		*p* = 0.498		*p* = 0.523		
	Male	200	2.83 ± 1.24	34	2.17 ± 0.98	0.004^*^
	Female	197	2.93 ± 1.74	38	2.34 ± 1.13	0.009^*^
Disease severity		*p* = 0.505				
	PANSS score (<90)	231	2.84 ± 1.40	72	2.26 ± 1.06	<0.001^**^
	PANSS score (>90)	166	2.94 ± 1.64	72	2.26 ± 1.06	<0.001^**^
EOS (×10^8^/L)		397	1.50 ± 0.83	72	1.23 ± 0.86	0.013^*^
IgA (mg/dL)		397	274.46 ± 75.86	72	259.56 ± 66.21	0.119

Inter-group comparisons were made using ANOVA, and the remaining comparisons using the *t*-test. “*” indicates a significant difference of *p* < 0.05, “**” indicates a significant difference of *p* < 0.001. IL-5, interleukin-5; EOS, eosinophils; IgA, immunoglobulin A; PANSS, Positive and Negative Syndrome Scale.

### 3.2 Elevated IL-5 Levels in the First-Episode Group, and Elevated Levels of Both IL-5 and EOS in the Recurrent/Chronic Group

No significant difference in the gender ratio was observed between the first-episode group, the recurrent/chronic group, and the healthy control group. However, the mean age of patients in the first-episode group was significantly younger than that of the other two groups (mean age: 23.5 years for the first-episode group, 49.5 years for the recurrent/chronic group, and 41.46 years for the healthy control group). Multi-group variance analysis showed that IL-5, EOS and IgA levels were significantly different between the groups (*p* < 0.05) (Table 2). However, post hoc comparisons revealed the IgA level was not significantly different between any two groups. The IL-5 level was significantly higher in both the first-episode and recurrent/chronic groups compared to the healthy control group, with no significant difference observed between the two patient groups. This suggests that elevated IL-5 levels may persist throughout the course of schizophrenia. Additionally, EOS levels were significantly higher in the recurrent/chronic group compared to the healthy control group, whereas no significant difference was found between the first-episode group and the healthy control group. The increased EOS level may therefore be related to the disease progression of schizophrenia, although an impact from the use of medication cannot be ruled out.

**Table 2.** **IL-5, EOS, and IgA levels in healthy individuals and in patients with first-episode schizophrenia and recurrent/chronic schizophrenia**.

	Healthy individuals	First-episode group	Recurrent/chronic group	*p*
Male [n (%)]	34 (47.22)	31 (54.39)	169 (49.71)	0.715
Female [n (%)]	38 (52.78)	26 (45.61)	171 (50.29)	
Age [M (QR) (Year)]	41.46 (25.12)	23.50 (8.50)	49.50 (27.50)	<0.001^**^
IL-5 (pg/mL)	2.26 ± 1.06	2.73 ± 1.22	2.91 ± 1.55	0.003^*^
EOS (×10^8^/L)	1.23 ± 0.86	1.41 ± 0.59	1.51 ± 0.87	0.032^*^
IgA (mg/dL)	259.56 ± 66.21	257.79 ± 63.22	279.14 ± 73.35	0.022^*^

Gender data were compared using the chi-square test, age data were compared using the Kruskal-Wallis test, and inter-group comparisons were analyzed using ANOVA. “*” indicates a significant difference of *p* < 0.05, “**” indicates a significant difference of *p* < 0.001.

### 3.3 The PANSS Score and IL-5 Levels Decreased in Patients With First-Episode Schizophrenia After Treatment

After two months of drug treatment, the PANSS scores and IL-5 levels all showed significant reductions in first-episode schizophrenia patients. No statistically significant differences were observed in the EOS and IgA levels before and after treatment (Table 3). Of the 57 patients, 37 showed a decrease in IL-5 level after treatment, 18 showed an increase and 2 showed no change, with a statistically significant difference (*p* < 0.001) observed in the decrease group and the increase group before and after treatment (Table 4). The aforementioned findings suggest that the elevated levels of IL-5 and EOS are correlated with the disease itself, rather than being attributable to the antipsychotic medications administered. However, the change in IL-5 varies among individuals, possibly due to the type of drug used and inter-individual differences.

**Table 3.** **Comparison of PANSS scores, IL-5, EOS and IgA levels in first-episode schizophrenia patients before and after treatment (n = 57)**.

	Before treatment	After treatment	*p*
PANSS Positive	23.73 ± 8.22	14.71 ± 5.22	<0.001**
PANSS Negative	19.23 ± 7.09	13.73 ± 5.84	<0.001**
PANSS General	43.73 ± 7.22	29.73 ± 6.62	<0.001**
IL-5 (pg/mL)	2.73 ± 1.22	2.18 ± 0.71	<0.001**
EOS (×10^8^/L)	1.41 ± 0.59	1.37 ± 0.49	0.349
IgA (mg/dL)	257.79 ± 63.22	247.02 ± 41.39	0.105

The paired *t*-test was used for comparisons; “**” indicates a significant difference of *p* < 0.001.

**Table 4.** **Comparison of the IL-5 level between groups of first-episode schizophrenia patients that showed either increased or decreased IL-5 after treatment (n = 55)**.

		Decreased IL-5 (n = 37)	Increased IL-5 (n = 18)
Gender	Male [n (%)]	22 (59.46%)	8 (44.44%)
	Female [n (%)]	15 (40.54%)	10 (55.56%)
	*p*	0.294	
IL-5 (pg/mL)	Before treatment	2.95 ± 1.34	2.33 ± 0.83
	After treatment	1.94 ± 0.60	2.66 ± 0.67
		Reduced 34.24%	Elevated 14.16%
	*p*	<0.001**	<0.001**

Gender data were compared with the chi-square test, and other comparisons were made using the paired *t*-test. “**” indicates a significant difference of *p* < 0.001.

### 3.4 Correlation Analyses of IL-5, EOS, IgA, Disease Duration and PANSS Scores in Patients With Schizophrenia

Correlation analysis revealed that, after adjusting for potential confounding factors such as age, gender, and disease status, IL-5 levels were significantly positively correlated with EOS levels (r = 0.338, *p* < 0.001). Furthermore, a weak positive correlation was observed between disease duration and EOS levels (r = 0.171, *p* < 0.05), while no significant correlations were observed for the other indicators (Table 5). In conclusion, the increase in EOS observed in schizophrenia patients may be associated with increased IL-5, and patients with longer disease duration may exhibit higher EOS levels. However, the weak correlation indicates that other influencing factors might also be involved.

**Table 5.** **Correlation analysis of IL-5, EOS, IgA, disease duration and PANSS scores in patients with schizophrenia (n = 397)**.

	PANSS Positive	PANSS Negative	PANSS General	IL-5	EOS	IgA	Disease duration
PANSS Positive	1	–0.042	0.145	0.167	0.131	0.166	0.178
PANSS Negative		1	–0.021	0.139	–0.097	0.093	0.146
PANSS General			1	0.156	0.141	0.144	0.159
IL-5				1	0.338^**^	0.183	0.141
EOS					1	0.098	0.171^*^
IgA						1	0.197
Disease duration							1

Correlation analysis was conducted using partial correlation analysis, *p* values were corrected by Bonferroni correction. “*” indicates a significant difference of *p* < 0.05, “**” indicates a significant difference of *p* < 0.001.

### 3.5 Predictive Value of IL-5 and EOS for Schizophrenia

**Fig. 1.**
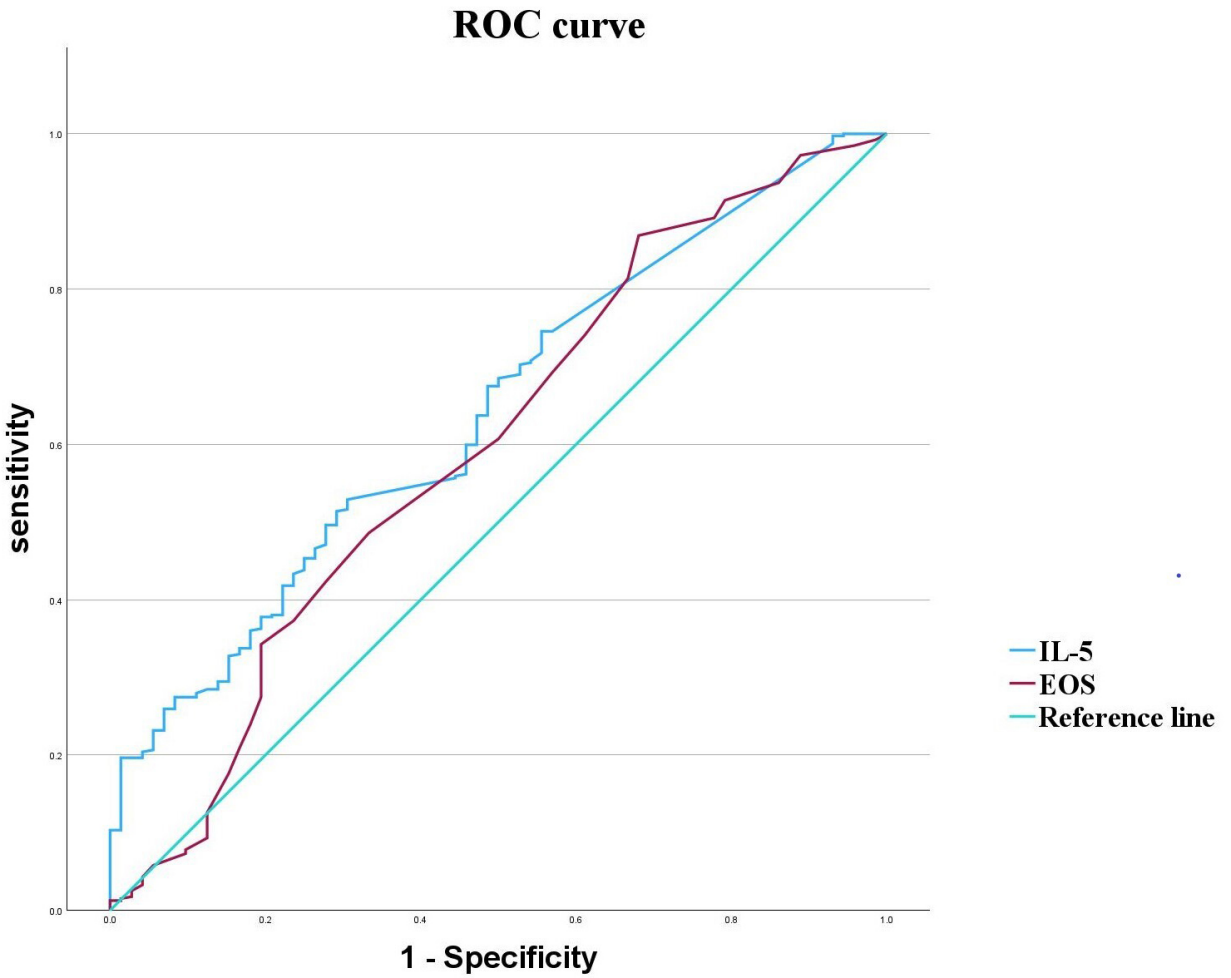
**ROC curves for IL-5 and EOS in schizophrenia**.

ROC curve analysis revealed that IL-5 and EOS exhibited area under the curve (AUC values) for schizophrenia of 0.641 and 0.596, respectively, with sensitivities of 52.9% and 86.9%, and specificities of 69.4% and 31.9%. The optimal cut-off points were determined to be 2.445 pg/mL for IL-5, and 0.75 × 10^8^/L for EOS (Table 6, Fig. 1). These findings indicate that EOS has relatively low specificity for predicting schizophrenia. IL-5 exhibited superior predictive performance for schizophrenia compared to EOS.

**Table 6.** **Performances of IL-5 and EOS in schizophrenia identification**.

	AUC	95% CI	Sensitivity (%)	Specificity (%)	Cut-off value	Youden’s index	*p*
IL-5	0.641	0.575–0.706	52.90	69.40	2.445 (pg/mL)	0.223	<0.001**
EOS	0.596	0.521–0.672	86.90	31.90	0.750 (×10^8^/L)	0.188	0.013*

“*” indicates a significant difference of *p* < 0.05, “**” indicates a significant difference of *p* < 0.001.

## 4. Discussion

An increasing number of studies have demonstrated that immune inflammation plays a significant role in the pathogenesis of schizophrenia. Inflammatory mediators can activate the tryptophan-kynurenine metabolic pathway to promote the synthesis of quinolinic acid and 3-hydroxykynurenine. This causes neurotoxicity in the central nervous system, thereby affecting adjacent neurons and nerve cells [29, 30]. A comparative review of serum/plasma biomarkers for differentiating schizophrenia from healthy individuals found that >70% of the potential markers are involved in inflammatory responses [31]. Inflammation may therefore be a consequence of schizophrenia, as well as a risk factor that contributes to its onset and progression.

### 4.1 Changes in the Level of IL-5 in the Serum of Patients With Schizophrenia

In the present study, IL-5 levels in patients with schizophrenia were significantly higher than those in healthy individuals, consistent with previous findings [32, 33]. However, no significant difference in IL-5 was observed among patients over 60 years old. This may be attributed to a decline in immune function in elderly individuals or to the use of medications such as non-steroidal anti-inflammatory drugs (NSAIDs) for other chronic diseases [17]. Both male and female patients exhibited significantly higher IL-5 levels than healthy controls, with no significant gender difference. However, female patients showed greater variability in the IL-5 level and a higher prevalence of abnormal levels than male patients. Other studies have also reported similar gender-related effects on cytokine levels in patients with schizophrenia, possibly due to the influence of menstrual cycles and sex hormones on cytokine concentrations in women [34, 35, 36], or it could be attributed to the differences in male and female genes, such as Female individuals carrying the homozygous T allele of *IL-8* gene rs1126647 polymorphism exhibit an increased susceptibility to paranoid schizophrenia [37].

Certain anti-psychotic drugs can cause eosinophilia in patients [38], and IL-5 promotes the growth, differentiation, and survival of EOS. Therefore, the potential impact of antipsychotic drugs on IL-5 levels cannot be excluded. To further investigate this effect, we compared IL-5 levels between first-episode and recurrent/chronic patient groups, as well as in first-episode schizophrenia patients before and after treatment. The findings indicate that elevated IL-5 and EOS levels are more likely associated with the disease itself rather than being induced by medication. Previous *in vitro* and *in vivo* studies have also demonstrated the anti-inflammatory effects of anti-psychotic drugs, with clozapine, chlorpromazine, haloperidol, aripiprazole and risperidone all shown to reduce the production of inflammatory cytokines [17, 18, 39, 40]. Notably, the inconsistent trend of IL-5 changes observed before and after treatment may be attributed to the type of drugs administered and individual variability among patients.

### 4.2 The Differences in IgA Levels in the Serum and Intestinal Mucosa of Patients With Schizophrenia

In the current study, no significant differences in serum IgA levels were observed between the different groups. However, animal studies have demonstrated that IL-5 can activate B cells in the large intestine of mice, leading to the induction of IgA+ B cells and the recruitment of EOS around the activated B cell areas, thereby enhancing IgA secretion in the large intestine [41]. Similarly, studies on schizophrenia have reported elevated gut IgA levels in patients with schizophrenia, which are negatively correlated with the richness of the gut microbiota [42]. The difference in IgA levels between blood and mucosa suggests that the effect of IL-5 on IgA may mainly be manifested in mucosal sites such as the intestine in schizophrenia.

### 4.3 The Role of EOS in the Progression of Schizophrenia

In the analysis of EOS levels, it was observed that the EOS levels in the relapse/chronic group were significantly higher compared to the healthy controls, a mild positive correlation was observed between EOS levels and the duration of the disease. It has been reported that the level of the EOS chemotactic factor Eotaxin-1/CCL11, which selectively recruits EOS to inflammatory sites, is significantly increased in patients with schizophrenia, and the severity of negative symptoms exhibits a positive correlation with the concentration of Eotaxin-1/CCL11 [25]. In addition, during the acute phase of schizophrenia, EOS levels are observed to temporarily decrease; following 6 weeks of treatment, their levels gradually return to normal; during the remission phase of schizophrenia, EOS levels may exhibit an increase. Changes in EOS counts are found to be negatively correlated with both the total score and the positive symptom subscore of the PANSS [43]. This is largely consistent with our observation results. However, we did not detect the transient decrease of EOS in the acute phase or its correlation with PANSS scores, which may be attributable to the grouping strategy and sample size. These findings indicate firstly that short-term drug treatment does not significantly affect EOS levels, although the potential influence of long-term medication remains uncertain; secondly fluctuations in EOS levels may correlate with the progression stage of schizophrenia.

Based on the known characteristics of EOS, it is hypothesized that they may contribute to two distinct roles in the pathogenesis and progression of schizophrenia. First, in patients with schizophrenia, overactivation of the 5-hydroxytryptamine 2A receptor (5-HT2A) triggers upregulation of the eosinophil chemotactic factor Eotaxin-1/CCL11. This upregulation facilitates the migration of eosinophils across the compromised blood-brain barrier to inflammatory sites within the brain, thereby induces degranulation, leading to the release of eosinophil peroxidase (EPO), eosinophil cationic protein (ECP), eosinophil-derived neurotoxin (EDN), and various cytokines, including IL-5, thereby exacerbating neuroinflammation and causing neuronal damage [25, 44, 45, 46, 47]. Additionally, EOS are involved in tissue repair and remodeling processes. T cells can regulate the expression of IL-4 in the decellularized nerve or acellular nerve allograft (ANA) environment by influencing EOS, thereby promoting the repair and regeneration of peripheral nerves [47, 48]. However, considering the irreversible nature of central neuronal damage in schizophrenia, this potential repair function may have limited clinical relevance.

IL-5, EOS, and IgA are not isolated entities but integral components of the body’s immune system. Studies have shown that patients with schizophrenia exhibit elevated levels of monocytes, neutrophils, and C-reactive protein (CRP). Additionally, various cytokines (e.g., monocyte chemoattractant protein-1, IL-1β, IL-5, IL-6, IL-9 and TNF-α) have been reported to be upregulated in schizophrenia [32, 33, 49, 50, 51]. The elevation of pro-inflammatory biomarkers such as IL-6, IL-1β, TNF-α, and CRP has been associated with cognitive decline, with this association being particularly pronounced in patients with deficit schizophrenia [52, 53]. However, it is important to highlight that following treatment, an increase in EOS levels coincides with a reduction in neutrophil counts, IL-6, and CRP levels, which aligns with the “eosinophil-driven recovery dawn” phenomenon observed during the resolution of bacterial inflammation. This indicates that EOS may play a significant role in the pathogenesis of schizophrenia, though whether this role is beneficial or detrimental remains to be elucidated through further research.

## 5. Limitations

IL-5 and EOS are significantly elevated in patients with schizophrenia and exhibit a certain degree of correlation. Although the ROC curve analysis demonstrated that IL-5 exhibited superior predictive ability for schizophrenia compared to EOS, when IL-5 is assessed as a standalone biomarker without integration with other cytokines, its diagnostic utility remains relatively restricted. Future studies should explore integrated analyses that incorporate cytokine profiles, cell surface markers, and broader functional outcome measures, or conduct more refined stratified analyses based on the optimal cut-off value of IL-5. Such approaches would contribute to a deeper understanding of the immune mechanisms underlying schizophrenia and improve the evaluation of the diagnostic significance of immune-related indicators.

The differences observed before and after treatment indicate that antipsychotic drugs exert a clear anti-inflammatory effect, suggesting that the elevated levels of IL-5 and EOS are associated with the disease rather than being caused by the medication. However, first-episode schizophrenia patients in our study primarily received medications such as risperidone, aripiprazole, olanzapine, and paliperidone upon admission. Due to poor patient compliance or adverse drug effects, some patients switched medications during treatment. There were instances of combined use of antidepressants, anxiolytics, and sedatives; therefore complicating the medication regimen and hence the interpretation of results. Moreover, differences may exist between first-line and second-line treatments for schizophrenia, and the effects of various drugs on IL-5, EOS, and IgA levels may not be entirely consistent. For example, patients taking clozapine exhibit lower IgA levels compared to those using other medications [54]. Furthermore, clozapine has a more pronounced inhibitory effect on neutrophils while also significantly increasing the level of EOS [38]. Therefore, assessing data from first-episode schizophrenia patients two months post-treatment presents certain limitations and the potential influence of adverse reactions resulting from long-term medication on the outcome cannot be excluded. Previous studies have demonstrated that conducting long-term follow-up research on patients with first-episode schizophrenia holds substantial significance [55]. Such research can not only identify risk factors associated with readmission and systematically evaluate the effects of medication changes on immune markers, but also offer an in-depth analysis of the potential roles that immune-related indicators, such as IL-5, EOS, and IgA, play in the pathogenesis and progression of schizophrenia.

## 6. Conclusions

Our findings indicate that in patients with schizophrenia, the levels of IL-5 and EOS were significantly elevated. The increase in EOS counts may be associated with elevated IL-5 levels, and patients with longer disease durations tended to exhibit higher EOS counts. Compared to EOS, IL-5 demonstrated greater predictive accuracy for schizophrenia; however, its diagnostic value as a standalone marker remains limited. IL-5 can be considered a key driver regulating the growth and differentiation of EOS, while EOS may function as effector cells in the body, exhibiting both pathogenic and reparative properties. Currently, the roles of IL-5, EOS, and IgA in the pathogenesis and progression of schizophrenia remain unclear. Further mechanistic studies are warranted. Potential approaches include incorporating additional relevant biomarkers and genetic analyses, conducting animal experiments, or performing long-term cohort studies to gain deeper insights into these mechanisms.

## Availability of Data and Materials

The data supporting the results of this study are available from the corresponding author upon reasonable request.
